# Improvement in progressive multifocal leukoencephalopathy after pembrolizumab‐induced immune reconstruction inflammatory syndrome in a patient with follicular lymphoma

**DOI:** 10.1002/jha2.56

**Published:** 2020-10-23

**Authors:** Inès Dufour, Thierry Duprez, Marie Wertz, Pascale Saussoy, Nathalie Ackermans, Souraya El Sankari, Vincent van Pesch, Eric Van Den Neste

**Affiliations:** ^1^ Department of Hematology Université catholique de Louvain, Cliniques universitaires Saint‐Luc Brussels Belgium; ^2^ Department of Radiology and Medical Imaging Université catholique de Louvain, Cliniques universitaires Saint‐Luc Brussels Belgium; ^3^ Department of Neurology Clinique Notre‐Dame de Grâce Gosselies Belgium; ^4^ Hematology Laboratory Université catholique de Louvain, Cliniques universitaires Saint‐Luc Brussels Belgium; ^5^ Department of Neurology Université catholique de Louvain, Cliniques universitaires Saint‐Luc Brussels Belgium

**Keywords:** Follicular lymphoma, immune reconstruction inflammatory syndrome, JC virus, pembrolizumab, progressive multifocal leukoencephalopathy

## Abstract

Progressive multifocal leukoencephalopathy (PML) may develop in follicular lymphoma patients treated with bendamustine‐rituximab. In this report, treatment with pembrolizumab successfully inhibited the clinical progression of PML by promoting radiologically demonstrated immune restoration inflammatory syndrome (IRIS), allowing complete clearance of the virus. These findings may further support the use of pembrolizumab in PML with special consideration for the potential occurrence of IRIS.

## 1 INTRODUCTION

In their recent publication, D'Alo et al [1], (2020 issue) thoroughly describe three cases of progressive multifocal leukoencephalopathy (PML) occurring in patients with follicular lymphoma treated with the combination of bendamustine and rituximab (BR). Deep and prolonged immunosuppression is a contributing factor for the onset of PML, a rare opportunistic brain infection caused by polyoma JC virus (JCV). So far, only scarce and divergent information is available on PML management. The disease is usually fatal unless immune function can be restored. We would like to share our single experience involving an additional patient with follicular lymphoma treated with BR who developed PML. His neurological status improved and CSF viral load was cleared following treatment with pembrolizumab, an immune checkpoint inhibitor (CPI) having triggered an immune restoration inflammatory syndrome (IRIS).

## 2 CASE PRESENTATION

A 67‐year‐old male, with extensive lymphadenopathies and a large retroperitoneal mass, was diagnosed in October 2016 with follicular lymphoma, histologically grade 2, stage IVB, with high‐risk Follicular Lymphoma International Prognostic Index (FLIPI) score and indication to treat according to the GELF criteria [2]. He achieved complete response to BR induction, followed by rituximab maintenance for 2 years. In September 2019, 8 months after termination of maintenance course, he complained from weakness and numbness in the right lower limb and developed progressive right‐sided hemiparesis, pyramidal syndrome, and speech disturbances. Sensitive testing showed right trimodal hypoesthesia.

**FIGURE 1:**
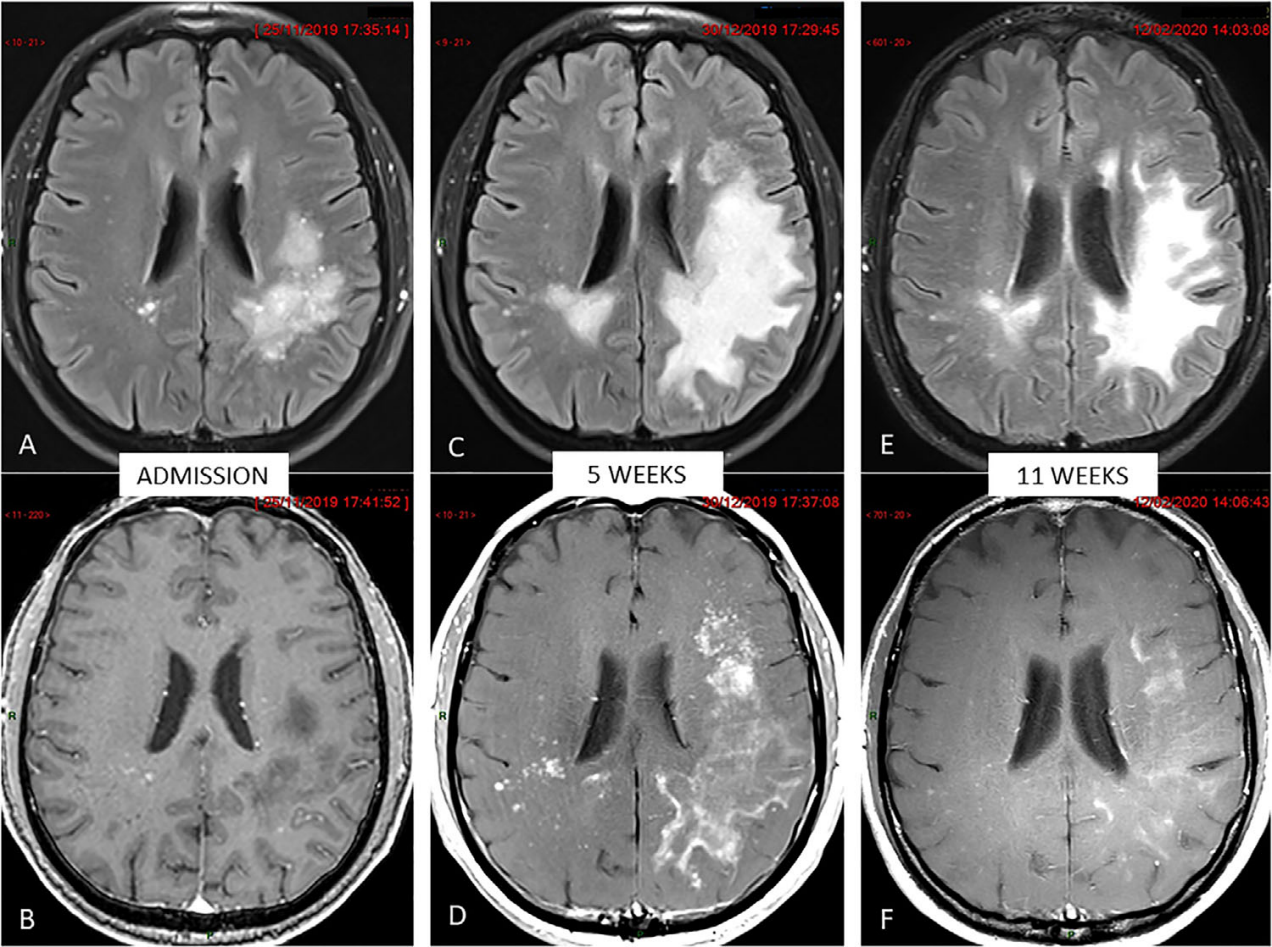
Brain magnetic resonance monitoring (all images in similar slice location). Upper row: Contrast‐enhanced FLAIR (fluid‐attenuated inversion recovery) views. Lower row: Contrast‐enhanced T1‐weighted views. **A** and **B**, Pretreatment baseline examination showing bilateral but asymmetric hyperintense changes in the parietal white matter on FLAIR image (A) appearing hypointense with only a few enhanced foci on post‐contrast image (B). **C** and **D**, Follow‐up examination 5 weeks later showing increase in size of damaged parenchyma on FLAIR image (C) together with dramatic increase of contrast‐enhancement featuring immune restoration (D). **E** and **F**, Follow‐up examination 11 weeks later showing moderate subsidence of FLAIR abnormalities (E) but strong decrease of areas of contrast enhancement (F) featuring the restoration of a competent brain‐blood barrier (BBB) after the inflammatory blaze seen in (D)

Brain magnetic resonance (MR) imaging revealed extensive parenchymal damage within the left parietal white matter disclosing T1 hypointensity, T2/fluid‐attenuated inversion recovery (FLAIR) hyperintensity, and only a few millimetric foci of contrast enhancement (Figure 1A,B). PCR analysis of the cerebrospinal fluid (CSF) detected 1438 DNA copies of JCV per milliliter. Immunophenotyping of peripheral blood showed a marked reduction of the CD4^+^ T‐cell count at 85/μL (normal range 630‐1400). B cells detected by CD19 were almost absent (6/μL, normal range 100‐410).

In order to enable immune recovery, pembrolizumab was initiated at a dose of 2 mg per kilogram of body weight every 4 weeks for four doses. Following the first cycle, the clinical status worsened, with onset of severe aphasia and the patient became wheelchair bound. Follow‐up MR examination 5 weeks after the first administration of pembrolizumab showed an increase in lesion size on FLAIR imaging, together with a dramatic increase in contrast enhancement consistent with immune restoration (Figure 1C,D). After the second cycle, the aphasia improved as the motor deficit in the lower limb, with a strength of 4/5 according to Medical Research Council (MRC) scale. Posttreatment MR demonstrated significant subsidence of the extent of the T2/FLAIR changes and a drastic decrease in contrast enhancement (Figure 1E,F). JCV was no longer detectable in the CSF. CD4+ T‐cell count remained low at 118/μL. Seven months after the diagnosis of PML, and 3 months after the last dose of pembrolizumab, neurologic symptoms continue to improve; the patient is progressively recovering his walking ability and performing activities of daily life independently.

## 3 DISCUSSION

The prognosis of patients with PML associated with an underlying hematologic cancer is poor. Reported mortality rates lie around 74‐100%, with a median survival of 2 months [3]. The pathogenesis of PML is characterized by an elective cytolysis of the oligodendrocyte glial cells by JCV resulting in rapidly extending demyelinating lesions in the absence of an immune response [4]. Although the mechanisms of controlling JCV infection are not yet completely understood, both humoral and cellular immune responses probably play a role [5]. Accordingly, the presence of JCV‐specific CD8^+^ and CD4^+^ T cells has been linked to the recovery from PML, while these cells were absent in cases of fatal outcome [6].

This case supports the concept that blocking the pathway of programmed death 1 (PD‐1) by CPI can potentially control JCV infection by triggering effective immune reconstitution. PD‐1 is present on the surface of T cells and acts as a negative regulator of immune responses. Interestingly, Tan et al showed that PD‐1 expression was elevated on total CD4^+^ and CD8^+^ T‐cells in PML patients when compared to healthy control subjects [7]. In the frame of chronic infection, PD‐1 expression may lead to impaired viral clearance, such as HIV and hepatitis B viruses [8]. PD‐1 induces T‐cell quiescence and inhibits T‐cell activation against virus, resulting in a viral latency [9]. Others have proposed that PD‐1 inhibitors may be an adjunctive therapy for chronic infections including HIV [9]. Pembrolizumab, a humanized antibody against PD‐1, induces downregulation of PD‐1 expression on lymphocytes, thereby increasing CD4^+^ and CD8^+^ activity against the JCV. This restored JCV‐specific cellular immune response, allowing a complete viral control [10]. Our patient presented an IRIS after the first infusion of CPI, of which “immune storm” resulted in an initial neurological worsening. Presumptively the overwhelming inflammatory response against JCV may have initially exacerbated symptoms, as it has been suggested in a patient with PML following lung transplantation and immunosuppressant dose reduction [11]. Others have showed that IRIS was concomitant with a decline in the level of PD‐1‐expressing circulating CD4^+^ and CD8^+^ T cells, occurring in some patients within 5 weeks after the first course of CPI [13]; Walter et al., 2019). In the literature, six of the 10 patients who received pembrolizumab for PML showed clinical improvement or stabilization, and one with transient imaging evidence of IRIS [10, 12].

Bendamustine is an alkylator with purine analogue properties resulting in lymphodepletion [14]. Profound CD4^+^ lymphopenia has been largely described in clinical studies with BR[15] with a very slow dynamic of CD4^+^ lymphocyte recovery after treatment and higher risk for opportunistic infections.

In conclusion, we report an additional patient with follicular lymphoma treated with BR who developed profound CD4^+^ lymphopenia and subsequent PML. The treatment with pembrolizumab successfully inhibited the progression of the disease by promoting the radiologically demonstrated immune restoration, allowing complete clearance of the virus. These findings may further support the use of pembrolizumab in PML with special consideration for the potential occurrence of an IRIS.

## CONFLICT OF INTEREST

The authors declare no conflict of interest.

## AUTHOR CONTRIBUTIONS

Inès Dufour, Eric Van Den Neste, Thierry Duprez, Marie Wertz, Pascale Saussoy, Nathalie Ackermans, Souraya El Sankari, and Vincent van Pesch contributed to patient management, data collection, and revised the paper. Inès Dufour and Eric Van Den Neste wrote the paper.

4

## Data Availability

The data that support the findings of this study are available on request from the corresponding author. The data are not publicly available due to privacy or ethical restrictions.
